# Assessment of Abdominal Aorta Balloon Occlusion Efficiency and Safety in Patients with Placenta Accreta Spectrum Disorder: A Systematic Review and Meta-Analysis

**DOI:** 10.3390/jcm15093400

**Published:** 2026-04-29

**Authors:** Meruyert Abdukassimova, Gulzhanat Aimagambetova, Milan Terzic, Altynshash Rakhat, Karlygash Togyzbayeva, Lyazzat Saidildina, Gauri Bapayeva

**Affiliations:** 1 Department of Surgery, School of Medicine, Nazarbayev University, 010000 Astana, Kazakhstanmilan.terzic@nu.edu.kz (M.T.); 2 Clinical Academic Department of Women’s Health, CF “University Medical Center”, 010000 Astana, Kazakhstangauri.bapayeva@gmail.com (G.B.); 3 School of Medicine, Nazarbayev University, 010000 Astana, Kazakhstan

**Keywords:** balloon occlusion of the aorta, abdominal aorta balloon occlusion, AABO, placenta accreta, placenta accreta syndrome, cesarean section

## Abstract

**Background/Objectives**: Placenta accreta spectrum (PAS) disorders are a major cause of life-threatening obstetric hemorrhage and frequently necessitate cesarean hysterectomy. Abdominal aorta balloon occlusion (AABO) has been increasingly adopted as a strategy to reduce intraoperative blood loss during cesarean section. This study aims to evaluate the effectiveness and safety of AABO during cesarean delivery in women with PAS disorders. **Materials and Methods**: A systematic review and meta-analysis of studies published in English from 2015 to April 2025 was conducted using the following databases: Embase, Scopus, PubMed, Google Scholar, and Web of Science. Articles that met inclusion criteria focused on human participants, original studies, female participants, and studied the efficacy of AABO on blood loss during cesarean delivery for PAS. Articles that were reviews, case reports, other occlusion procedures, and animal studies were excluded. Risk of bias was evaluated using the Newcastle-Ottawa Scale. **Results**: Twenty-four studies comprising 1958 cesarean deliveries with AABO and 1791 without AABO met the inclusion criteria. Data on blood loss, transfusion, hysterectomy, maternal complications, and neonatal outcomes were extracted, synthesized, and analyzed. The majority of studies (91.6%) applied the balloon at the infrarenal level. Cesarean delivery with AABO resulted in substantially lower mean blood loss (1231 ± 688 mL vs. 2253 ± 857 mL, *p* < 0.001) and reduced requirements for blood transfusion compared with cesarean delivery alone. Hysterectomy rates were threefold lower with AABO (7.8% vs. 25.8%, *p* < 0.001), and the incidence of hemorrhagic shock and re-laparotomy were markedly reduced. Complications associated with AABO, including lower limb arterial thrombosis and fever, were uncommon and generally manageable. **Conclusions**: AABO during cesarean delivery for PAS disorders is associated with reduced intraoperative blood loss, lower transfusion requirements, and decreased hysterectomy rates, suggesting improved maternal hemodynamic stability. Although the procedure is generally safe, vigilance for vascular and thromboembolic complications is essential, and preventive strategies should be incorporated into perioperative care. These findings support the integration of AABO into multidisciplinary management protocols for women with PAS disorders. Future randomized prospective studies should be performed to improve patient selection criteria, standardize the protocols, and further evaluate the long-term maternal and neonatal safety/outcomes of the procedure.

## 1. Introduction

Abnormal placentation, including placenta accreta spectrum (PAS) disorder, presents a dangerous complication of pregnancy and labor [1]. Depending on the depth of placental invasion into the uterine wall, it is classified into placenta accreta, increta, and percreta [2,3,4]. Risk of developing PAS disorders has been associated with a previous cesarean section(s), advanced maternal age, in vitro fertilization (IVF), previous intrauterine manipulations, inborn uterine anomalies, and multiparity [2,5,6]. With the worldwide tendency to delay childbearing, increased use of IVF, and the increasing rate of cesarean delivery, the rate of PAS disorders is rising [5,6].

Surgical delivery for patients with PAS disorder can be life-threatening, with a high risk of asphyxia for a newborn and morbid hemorrhage for the mother, especially if undiagnosed during pregnancy [7]. Proper diagnosis of PAS disorder beforehand allows for planned surgery with preordered blood components, an autologous blood transfusion device, additional surgical team members on call, as well as planned use of additional techniques for hemorrhage control [7,8,9]. Many institutions still prefer direct hysterectomy over the risk of massive hemorrhage, which results in infertility and has a major psychological impact on a patient [4,8,10].

**Figure 1:**
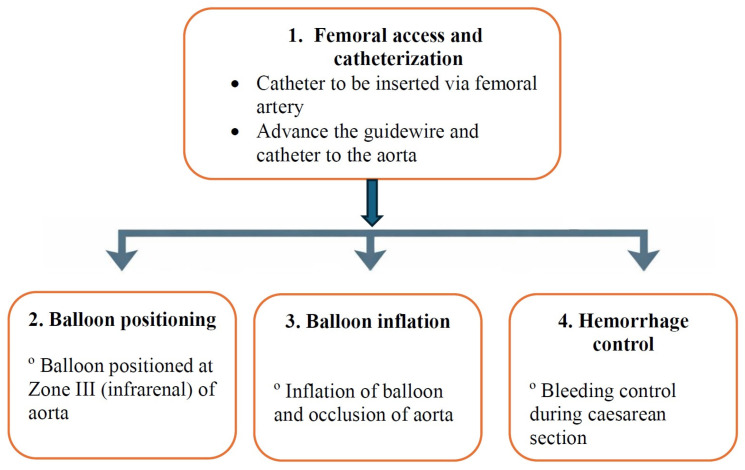
Schematic picture of the AABO procedure.

Different interventional techniques are currently available for hemorrhage control, such as uterine artery embolization (UAE), internal iliac artery balloon occlusion (IIABO), and, recently, abdominal aorta balloon occlusion (AABO) has been applied for obstetric emergencies [1,9]. Abdominal aorta balloon occlusion is an interventional radiology technique that allows for better hemostasis due to blockage of more collateral blood vessels in comparison to the internal iliac arteries, as well as time reduction for the placement of one balloon versus two into two iliac vessels. There are numerous studies that report the application of the AABO procedure and find it superior to other hemostasis techniques [4,5,10]. This procedure has been initially applied to trauma patients to reduce massive hemorrhages. In obstetrics, for performing AABO, a balloon catheter is inserted into Zone III of the aorta (the infrarenal part) for intraoperative bleeding control (Figure 1) [1].

The utilization of AABO in the obstetric setting has shown promising results, and it is found to be an effective method for reducing intraoperative blood loss and blood transfusion volume at the cesarean section in cases of morbidly adherent placenta [1,4]. However, the variability of technique and outcome measures among studies necessitates the need for the synthesis of existing data on the topic. This systematic review and meta-analysis aim to assess the existing research on the use of AABO in patients with PAS disorder, with an emphasis on the reduction in blood loss and associated risks.

## 2. Material and Methods

### 2.1. Study Registration and Methodological Standards

The study was registered in the International Prospective Register of Systematic Reviews (PROSPERO) on 14 December 2024, with a registration code of CRD42024621871. The study was conducted following the Preferred Reporting Items for Systematic reviews and Meta-Analyses (PRISMA) guideline (https://www.prisma-statement.org/ accessed on 12 April 2026).

### 2.2. Information Sources and Search Strategy

The systematic electronic literature search was followed by manual searching of the literature to identify studies by examining the reference lists of relevant studies and previous reviews to ensure that all potentially eligible publications were identified. The following databases were used: Embase, Scopus, PubMed, Google Scholar, and Web of Science. Research papers involving human subjects, investigating abdominal aorta balloon occlusion, and published in English online for the past 10 years, from 2015 to April 2025, were included in the study. The search was performed using keywords and medical subject headings (MeSH) unique identifiers, if available. The following keywords and keywords combinations were applied: Balloon occlusion (MeSH Unique ID: D021721), REBOA, Aortic balloon, Aortic vascular control, Prophylactic Resuscitative Endovascular Balloon Occlusion of the Aorta, Intra-aortic balloon, Aortic occlusion, Abdominal aorta balloon occlusion, AABO, ABO, Prophylactic aortic balloon occlusion, PABO, PAABO, Aortic vascular balloon control, Aortic vascular occlusion, Placenta (MeSH Unique ID: D010920), Placenta previa (MeSH Unique ID: D010923), Placenta accreta (MeSH Unique ID: D010921), Placenta accreta syndrome, PAS, Morbidly adherent placenta, MAP, abnormally invasive placenta, AIP, Placenta abnormality syndrome. The Boolean search strategy and search threads are presented in Supplementary Table S1. Abstracts, case series and reports, conference papers, letters to the editor, or commentaries, as well as papers published in languages other than English, were excluded. Only studies published in English were included in the review. This was to ensure consistency in methodology and accuracy of data extraction. We acknowledge that this may have resulted in the exclusion of some relevant reports and thus is a potential source of language bias.

Titles and abstracts of studies found through the search process were reviewed independently by two authors to determine their relevance to the goals of this study. Full texts of studies, which met the eligibility criteria, were obtained and evaluated separately by all four team members. Any disagreements about whether certain studies met the inclusion criteria were settled through group discussion among all team members.

### 2.3. Eligibility Criteria and PICO Statement

The articles that met the following eligibility criteria were included in the study: (1) human subject research; (2) research articles; (3) studies involving women; (4) studies assessing the effect of abdominal aorta balloon occlusion on blood loss during cesarean section for placenta accreta spectrum disorder. The following exclusion criteria were applied: (1) reviews and case reports, (2) irrelevance to abdominal aorta balloon occlusion (discussing other large vessel occlusion methods), and (3) animal model studies. Abstracts without full and clear information about the required criteria were excluded without further review. Population, Intervention, Comparison, Outcomes (PICO) statement: In women with PAS disorder—placenta accreta, increta, percreta (P), did the aortic balloon occlusion for hemorrhage control during cesarean section (I), compared with cesarean delivery in PAS patients without use of AABO (C), decrease blood loss during surgery (O)?

The following main outcomes were investigated: primary (mean blood loss, hemorrhagic shock, amount of blood transfusion, rates of hysterectomy and re-laparotomy) and secondary (bladder/ureter injury; intensive care unit stay/days; neonatal outcomes—birth weight, APGAR score, NICU admission, neonatal complications, and mortality).

### 2.4. Data Collection and Synthesis

The search was narrowed by using “Abdominal aorta balloon occlusion OR AABO AND Placenta accreta spectrum OR PAS”, “Aortic balloon occlusion AND Morbidly adherent placenta OR MAP”, “Intra-aortic balloon AND Placenta accreta spectrum OR PAS” (Supplementary Table S1). The following data were retrieved from the analyzed studies: author, year of publication, study origin, type, number of study participants, localization of balloon, blood loss volume, hysterectomy, red blood cells (RBC) transfusion amount, intensive care unit (ICU) admission, length of hospital stay (LHS), and additional hemostatic procedures performed.

### 2.5. Assessment of Risk of Bias

All studies included in the systematic review and meta-analysis were autonomously revised for inclusion eligibility by three reviewers (M.A., G.B., and M.T.). Any discrepancies in the evaluation of articles were resolved through the team members’ discussion (M.A., A.R., M.T., K.T., L.S., and G.B.). The risk of bias was evaluated according to guidelines to identify any deviations from planned interventions, outcome criteria measurement, missing outcome data, and the reported result selection. All studies were evaluated using the Newcastle–Ottawa Scale (NOS) for non-randomized studies [11]. The scale assesses selection (0–4 points), comparability (0–2 points), and outcome/exposure assessment (0–3 points), with scores ranging between 0 and 9. Based on the assessment, a low risk of bias was considered in studies with 7–9 points, a moderate risk with 5–6 points, and a high risk of bias with ≤4 points. In addition to the NOS evaluation, the certainty of evidence was also evaluated according to the principles of the Grading of Recommendations, Assessment, Development, and Evaluation (GRADE) framework, adapted for non-randomized observational studies. The evaluation took into account the limitations of the study design, the consistency of results across studies, the directness of the evidence, the precision of the outcome measures, and the risk of publication bias. For the core outcomes of blood loss, transfusion requirement, hysterectomy rate, and maternal complications, the confidence in the evidence was considered to be moderate to high for consistent results and low to moderate for less consistent results.

### 2.6. Ethics Statement

This study did not directly involve any human or animal data. Therefore, considering the nature of this study, a systematic review and meta-analysis, ethical approval, and informed consent are not required. Moreover, original research reports included in the analysis were checked for agreement with the ethical standards professed by the Helsinki Declaration.

### 2.7. Data Analysis

Descriptive statistical analysis was performed to compare the primary outcomes between cases and controls in the identified studies. A value of *p* < 0.05 was considered statistically significant. In addition, meta-analyses were conducted to pool effect sizes across studies and assess the overall consistency and robustness of observed outcomes. Separate meta-analyses were carried out for each primary outcome, namely intraoperative blood loss and hysterectomy rate. A random-effects model (REML) was applied because the included studies differed in several aspects, including study design, sample size, patient characteristics, and procedural protocols, which could contribute to variation in the underlying effect estimates. Potential sources of heterogeneity were further explored using meta-regression with pre-specified moderators, including total sample size, year of publication, intraoperative blood loss, and the baseline hysterectomy rate in the control group.

The study employed Stata/MP version 19.5 (StataCorp LLC, College Station, TX, USA) to conduct all analyses, which included pooled effect size estimation, heterogeneity assessment, meta-regression, and p-value calculation.

## 3. Results

### 3.1. Study Identification and Selection

**Figure 2:**
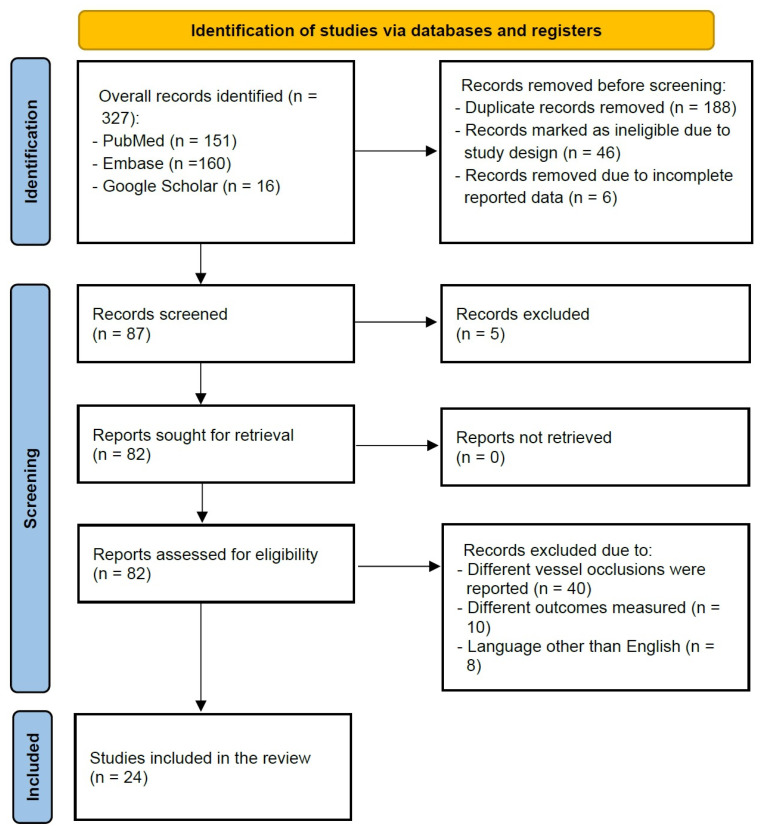
Data collection flow chart.

The initial screening on Embase, PubMed, Medline, Scopus, and Web of Science databases identified 327 articles (Figure 2). Out of all articles, 240 papers were excluded due to ineligibility (duplicated studies, study design not fulfilling the inclusion criteria, incomplete data reporting, etc.). The remaining 87 articles were assessed for eligibility based on the abstracts/full texts, and 63 articles were excluded because different vessel occlusions were reported, and different outcomes were measured. Finally, only 24 studies fulfilled the inclusion and exclusion criteria [2,3,4,5,6,8,9,12,13,14,15,16,17,18,19,20,21,22,23,24,25,26,27,28] and thus were selected for subsequent analysis (Figure 2; Table 1, Table 2 and Table 3).

The included studies reported data for 1958 cases (patients who underwent cesarean section with AABO) and 1791 cases (patients who underwent cesarean section alone). All studies included in the analysis are retrospective. The majority of them are published in indexed clinical journals with impact factors ranging from moderate to high. Moreover, more than 85% of studies reported positive maternal outcomes.

### 3.2. Study Outcomes

The study outcomes are compared and summarized in Table 1. The PAS subtypes were analyzed together due to data limitations in the included studies. The data for hemorrhagic shock and transfusion were recorded and analyzed as reported by the original studies, without standardized definitions/units across studies.

**Table 1:** Primary and secondary outcomes summary.

Outcome	AABO (n = 1958)	Control (n = 1791)	Relative Risk	Estimated Significance
Mean blood loss	1231 ± 688 mL	2253 ± 857 mL	45%	*p* < 0.001
Hysterectomy	7.76% (152/1958)	25.79% (462/1791)	0.30	*p* < 0.001
Hemorrhagic shock	3.8%	14.6%	0.26	*p* < 0.001
Re-laparotomy	2.8%	8.7%	0.32	*p* < 0.001
Neonatal outcomes	No difference	No difference	-	Not applicable

The primary outcomes were significantly different among groups (mean blood loss, amount of blood transfusion, hemorrhagic shock, rates of hysterectomy and re-laparotomy).

#### 3.2.1. Effectiveness of Cesarean Section with AABO

A comparison of the effectiveness of cesarean section with AABO use vs. cesarean section alone is presented in Table 2. Most studies (95.83%) were conducted in China, and only one study (4.16%) reported data from an Israeli study. Out of 24 studies analyzed, 91.6% reported the infrarenal level of balloon application, 4.16% performed Zone II balloon occlusion, and 4.16% (one study) did not report the level of aorta occlusion (Table 2). Mean blood loss among patients undergoing cesarean section with AABO was 1231.42 ± 688, compared with 2252.53 ± 857.06 in patients undergoing cesarean section without AABO (Table 2). Overall, in studies, which, together with cesarean section applied AABO, 7.76% of cases (152 out of 1958 cases) were completed with hysterectomy, while among patients with cesarean section without AABO, 25.79% of cases (462 out of 1791) were completed with hysterectomy. Blood transfusion demand was higher in patients who underwent cesarean section without AABO. 

All 24 studies reported that aortic balloon occlusion during cesarean section for PAS disorder is a safe and effective approach to improve maternal outcomes.

**Table 2:** Comparison of cesarean section outcomes among cases (cesarean section and AABO) vs. controls (cesarean section alone).

**Author, Year**	**Country**	**Study Design**	**Number of Patients**		**Localization of the Balloon**	**Blood Loss, mL**		Hysterectomy, n (%)		RBC Transfusion Amount		ICU Admission, n (%)		Length of Hospital Stay, Days		Additional Hemostatic Procedures		Conclusion
			**Case**	**Control**		**Case**	**Control**	Case	Control	Case	Control	Case	Control	Case	Control	Case	Control	
Chen et al., 2016 [5]	China	Retrospective	20	23	Infrarenal	1155.0 ± 393.7	2017.4 ± 874.7	4 (20) resection of uterus	12 (52.2)	580.0 ± 327.0 mL	1017.4 ± 635.8 mL	not reported	not reported	6.5 ± 1.2	7.7 ± 3.1	Uerotonics, hemorrhage area suturing, uterine artery ligation, ribbon gauze uterine cavity filling	Uterotonics, hemorrhage area suturing, uterine artery ligation, ribbon gauze uterine cavity filling	The balloon occlusion of the lower abdominal aorta seems effective in reducing PPH and blood transfusion. It is effective in decreasing the risk of hysterectomy without harming the newborns. Intraoperative and postoperative blood loss, RBC transfusion, and uterus resection are lower in the balloon group. Uterine cavity ribbon gauze filling is higher in the balloon group.
Cui et al., 2017 [2]	China	Retrospective	38	31	Infrarenal	1560.5 ± 1278.8	2145.2 ± 1160.1	2 (5)	3 (10)	3.6 ± 3.3 units	4.4 ± 3.5 units	38	31	8.5 ± 3.6	6.9 ± 1.5	Uterotonics, uterine artery embolization, and “other methods of hemostasis.”	Uterotonics, local suture ligation, uterine artery ligation, uterine packing	PBOAA reduced bleeding after cesarean delivery for women with abnormal placentation. No difference in maternal and neonatal demographics, EBL lower in PBOAA, no difference in units of blood transfused, no difference in LHS. Note the difference b/w placenta accreta/increta with percreta, percreta + balloon—show no difference with control in EBL
Duan et al., 2018 [10]	China	Retrospective	22	23	Infrarenal	597 ± 359	2687 ± 575	0	7 (30.4)	498 ± 195 (blood transfusion, not specified if RBC)	2390 ± 789 (blood transfusion, not specified if RBC)	0	2 (8.7)	5.8 ± 2.3	9.6 ± 3.9	UAE, UA ligation, uterine cavity stuffing	UAE, UA ligation, uterine cavity stuffing	Intermittent aortic balloon occlusion may control PPH in pregnancies complicated by placenta accreta and improve postop conditions. Balloon significantly reduces EBL, transfusion, length of surgery, and less patient required uterine cavity stuffing, UAE, UA ligation, shorter LHS
Huo et al., 2021 [6]	China	Retrospective	12	16	Infrarenal	3762.50 ± 3728.33	2831.25 ± 1906.03	3 (25)	2 (12.5)	10.66 ± 11.97 units	8.00 ± 5.84 units	7 (58.3)	8 (50)	8.25 ± 5.37	6.75 ± 2.57	not reported	Not reported	Authors advocate for AABO, but bleeding and transfusion were higher for AABO
Ioscovich et al., 2023 [8]	Israel	Retrospective cohort	10	11	Infrarenal	1060 ± 296.64	4400 ± 2787.0	3 (30)	10 (90.9)	median of RBC units—1 unit	median of PRBC units— 4 (0–9) units	0	5 (45)	not reported	not reported	not reported	Not reported	Balloon occlusion of aorta is safe and effective for MAP preventing massive bleeding, reducing rate of hysterectomy, and improving patient outcome
Li et al., 2018 [9]	China	Retrospective	24	32	Infrarenal	1600.00 ± 1185.785	2032.81 ± 1881.258	2 (8.3)	16 (50)	5.83 ± 3.754 units of PRBC	7.39 ± 3.794 units	9 (37.5)	9 (28.1)	8.08 ± 3.810	9.25 ± 5.388	UA ligation, uterine cavity filling with ribbon gauze or Bakri balloon tamponade	UA ligation, uterine cavity filling with ribbon gauze or Bakri balloon tamponade	Balloon occlusion of aorta is effective in reducing number of hysterectomies, but no difference in other characteristics. It is found to be a safe method.
Liu et al., 2021 [12]	China	Retrospective cohort	168	106	Infrarenal	931.02 ± 440.75	2068.30 ± 759.95	23 (13.7)	30 (28.3)	RBC suspension mL 374.76 ± 456.48	1076.47 ± 838.48	8 (4.8)	11 (10.4)	4.22 ± 1.636	4.51 ± 2.319	Not reported	B-Lynch suture, UAE, uterine packing, UA ligation,	AABO is a safe and effective method, reducing blood loss volume and transfusion volume.
Liu et al., 2022 [13]	China	Retrospective case–control	40	40	Zone 2 aorta (middle abdominal aorta)—at level between left and right renal arteries	811.75 ± 299.93	1529.75 ± 808.01	0	6 (15)	0 units (0–2)	2 units (0–4)	not reported	not reported	not reported	not reported	Blocking of uterine cervix with elastic tourniquet, UAE, ovarian AE, contractile drug injection, local suture ligations, UA ligation, B-Lynch	Contractile drug injection, local suture ligations, UA ligation, B-Lynch	Zone 2 abdominal aorta is safe when the single occlusion time is limited to 15 min, reducing blood loss, transfusion requirements, and hysterectomy rates.
Lu et al., 2021 [14]	China	Retrospective	66	194	Not reported	1000 (600–1800)	1800 (800–3000)	0	8 (4.1)	4 (4–8) units of PRBC	4 (4–10)	1 (1.5)	3 (1.5)	5 (4–7)	5 (4–7)	B-Lynch, UA ligation, tourniquet binding lower uterine segment	B-Lynch, UA ligation, tourniquet binding lower uterine segment	Planned BPAA significantly minimized intaoperative blood loss, the need for B-lynch suture, and RBC transfusion.
Luo et al., 2021 [16]	China	Retrospective case–control	162	133	Infrarenal	1239.5 ± 752.8	1728.6 ± 985.4	24 (14.8)	34(25.6)	5.5 ± 3.2 units	9.5 ± 4.6 units	27 (16.6)	37 (27.8)	6.5 ± 2.8	7.1 ± 3.2	UAE if bleeding after catheter removal	Not reported	EBL lower, RBC transfusion lower, shorter operation time, less decrease in hemoglobin, preserving fertility in the balloon group.
Luo et al., 2022 [15]	China	Retrospective case–control	30	34	Infrarenal	1000 ± 663	1768 ± 1100	2 (6.7)	5 (14.7)	600 ± 641 mL	1053 ± 1083 mL	not reported	not reported	8.5 ± 2.9	9.2 ± 2.9	Tourniquet binding of LUS, UAE	Tourniquet binding of LUS, UAE	AABO with a tourniquet could improve outcomes and reduce complications.
Mei et al., 2022 [17]	China	Retrospective observational	31	15	Infrarenal	2000 (550–3100)	3000 (2200–4500)	9 (29)	2 (13.3)	4 (2–8) units of RBC	8 (6–12) units	not reported	not reported	6 (5–8)	7 (7–7)	Not reported	Not reported	Lower EBL with AABO.
Sun et al., 2018 [18]	China	Retrospective	19	12	Infrarenal	mean 1200 mL (range 350–9000)	3150 mL (range 1500–8000)	4 (21.1)	6 (50)	800 (0–6050) mL	1950 (1000–6800)	not reported	not reported	7.0 (range 3–13)	6.5 (range 5–15)	Uterotonics, UA ligation	Uterotonics injection, UA ligation	PBOAA is a safe and effective method to reduce EBL, save the uterus, and save life from severe complications.
Wang et al., 2017 [19]	China	Prospective cohort	10	33	Infrarenal	median 1000 mL (1000–1625)	2000 mL (1000–3000)	7 (70)	21 (63.3)	median 1100 mL (800–1800) RBC	2000 mL (1400–2900)	not reported	not reported	not reported	not reported	“Conservative methods”	“Conservative methods”	AABO is a safe and effective method to control blood loss and blood transfusion in PAS.
Wang et al., 2022 [20]	China	Retrospective cohort	276	554	Infrarenal	1306.10 ± 71.39	2121.73 ± 121.24	3 (1.1)	84 (15.2)	not reported	not reported	not reported	not reported	not reported	not reported	Tourniquet compression, folding suture, vessel ligation, uterine packing	Tourniquet compression, folding suture, vessel ligation, uterine packing	AABO was associated with lower blood loss, rate of hysterectomy, repeated surgery, and blood transfusion. Also compared results by year, with each year improving outcomes.
Wang et al., 2023 [21]	China	Retrospective case–control	24	21	Infrarenal	1433.33 ± 1037.92	1474.29 ± 36.73	5 (20.8)	3 (14.3)	3.25 (6) units	0 (4) units	2 (8.3)	0	6.5 ± 2.8	5.0 ± 1.76	UAE	UAE	No difference in EBL, longer operation time with AABO, more RBC transfusions in balloon group, longer hospital stay, and more complications in balloon group.
Wu et al., 2016 [22]	China	Retrospective cohort	230	38	Infrarenal	921 ± 199	2790 ± 335	0 (0)	3 (7.89)	422 ± 58	1580 ± 67	3 (1.3)	5 (13.5)	5.1 ± 0.8	6.7 ± 1.0	Uterotonics, oversewing placental bed, uterine tamponade, UA ligation, UAE	Uterotonics, oversewing placental bed, uterine tamponade, UA ligation, UAE	AABO is a safe and effective method that reduces the hysterectomy rate.
Xie et al., 2017 [23]	China	Retrospective case–control	30	41	Infrarenal	961 ± 784	1560 ± 1353	5 (16.7)	10 (24.4)	400 (0–800) mL	400 (400–1200) mL PRBC	not reported	not reported	not reported	not reported	UAE	UAE	AABO is a safe and effective method for reducing EBL, reduce hysterectomy rate without obvious maternal and neonatal adverse outcomes.
Ye et al., 2023 [24]	China	Retrospective cohort	278	86	Infrarenal	1370 ± 752	3536.8 ± 1383.2	14 (5.04)	63 (73.26)	3.0 ± 4.0 units	13.8 ± 6.9 un	168 (60.4)	67 (77.9)	not reported	not reported	Parallel traversal compressing suture technique, UAE	Tourniquet compression around the cervix	AABO can reduce intraoperative hemorrhage in AIP patients. The next step is to identify associated risks and better define inclusion criteria for AABO use for better outcomes.
Yin et al., 2022 [25]	China	Retrospective	68	88	Infrarenal	1604.56 ± 1481.37	2078.32 ± 1822.87	12 (17.65)	23 (26.14)	3.96 ± 6.00 units of RBC	5.55 ± 7.13 units	not reported	not reported	7.66 ± 4.42 d	6.51 ± 2.82 days	Uterine compression sutures, intrauterine gauze packing, intrauterine balloon tamponade, UA ligation	Uterine compression sutures, intrauterine gauze packing, intrauterine balloon tamponade, UA ligation	AABO is safe and effective approach to improve maternal outcomes with PAS.
Zeng et al., 2017 [26]	China	Retrospective cohort	48	38	Infrarenal	1467.71 ± 1075.77	2218.42 ± 1572.2	2 (4.2)	9 (23.7)	5.42 ± 4.95 units	9.29 ± 7.59 units	3 (6.3)	10 (26.3)	7.25 ± 1.06	8.16 ± 1.50	Uterotonic drugs, Bakri tamponade, UAE, ligation of pelvic arteries, B-Lynch	Uterotonic drugs, Bakri tamponade, UAE, ligation of pelvic arteries, B-Lynch	Safe and effective method for placenta INCRETA (they have separate tables for percreta that we did not use, and also no statistically significant difference in percreta cases).
Zhao et al., 2024 [27]	China	Retrospective cohort	118	22	Infrarenal	980 ± 230.15	1105.26 ± 248.27	0 (0)	2 (9.09)	425.38 ± 109.15	503.24 ± 108.27	not reported	not reported	10.09 ± 3.95	9.55 ± 5.79	Not reported	Local sutures, UA ligation, uterine cavity tamponade and other methods	AABO reduces EBL, hysterectomy, need for transfusion, and related complications.
Zheng et al., 2019 [3]	China	Retrospective case–control	102	68	Infrarenal	600 (400–1000)	1000 (650–2000)	17 (16.7)	17 (25)	300 (0–700) mL PRBC	400 (0–1400) mL	17 (16.7)	15 (22.1)	6 ± 2.8	6.1 ± 3.2	Uterotonics, hemostatic sutures, UA ligation, uterine packing, UAE	Uterotonics, hemostatic sutures, UA ligation, uterine packing, UAE	AABO effective method, but should be reserved for very well antenatally diagnosed cases due to the high risk of thrombotic complications. Cesarean hysterectomy still remains best option for percreta cases.
Zheng et al., 2022 [28]	China	Retrospective case–control	132	132	Infrarenal	1804.96 ± 1680.45	3017.75 ± 1959.84	11 (8.3)	86 (65.2)	not reported	not reported	not reported	not reported	not reported	not reported	Tourniquet compression, folding suture, vessel ligation, uterine packing	Tourniquet compression, folding suture, vessel ligation, uterine packing	AABO can significantly reduce blood loss, hysterectomies, and repeat surgeries.

Table footnotes: AABO—abdominal aorta balloon occlusion; EBL—estimated blood loss; LHS—length of hospital stay; MAP—morbidly adherent placenta; RBC—red blood cells; PBOAA—prophylactic balloon occlusion of abdominal aorta; PPH—postpartum hemorrhage; BPAA—balloon placement in abdominal aorta; UA—uterine artery, UAE—uterine artery embolization.

**Figure 3:**
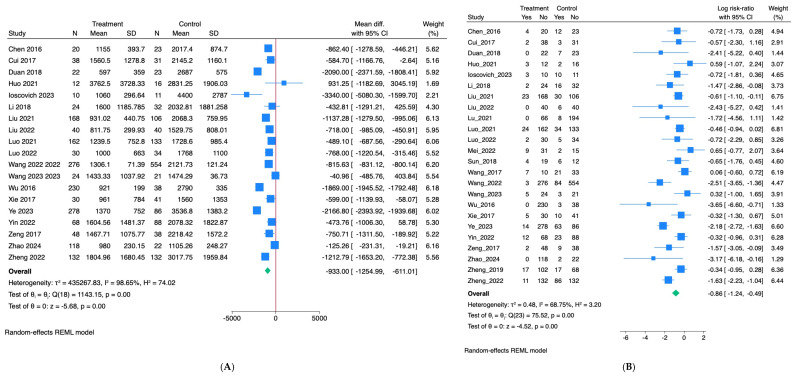
Forest plots of primary outcomes (AABO vs. control), random-effects REML model. Figure legend: (**A**). intraoperative blood loss (AABO vs. control); (**B**). hysterectomy (AABO vs. control). [2,3,4,5,6,8,9,10,13,14,15,16,17,18,19,20,21,22,23,24,25,26,27,28]. Blue squares (with 95% CIs) indicate individual study effects; the green diamond indicates the pooled effect.

**Figure 4:**
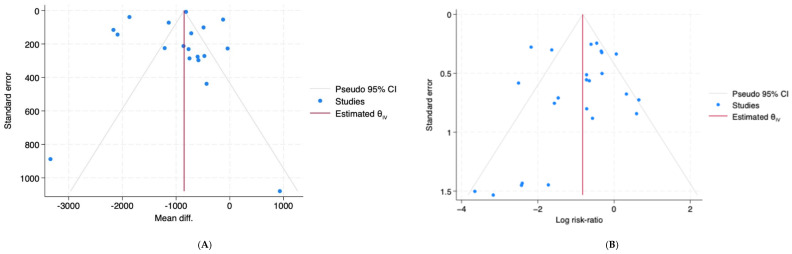
Funnel plots for primary outcomes (log risk ratio vs. standard error). Figure legend: (**A**). blood loss outcome (mean difference vs. standard error); (**B**). hysterectomy outcome (log risk ratio vs. standard error).

A meta-analysis was conducted separately for intraoperative blood loss rates and hysterectomy to assess whether AABO reduces these outcomes compared with cesarean section alone (Figure 3 and Figure 4). First, for intraoperative blood loss, 19 out of the 24 identified studies reported mean ± SD values and were included in the quantitative analysis. The remaining five studies were excluded from the meta-analysis because they reported blood loss as median or range, or did not provide standard deviations, making the calculation of effect sizes and pooling impossible.

The pooled results indicated that patients who underwent AABO lost, on average, 933 mL less blood during surgery compared to those who did not receive AABO (95% CI: 611 to 1255 mL less; *p* < 0.001), indicating a significant reduction in intraoperative blood loss. Despite this, heterogeneity across studies was very high (I^2^ = 98.7%), reflecting substantial variability in reported outcomes (Figure 3A).

Most studies placed the balloon infrarenally, but this factor did not explain the differences between the studies (*p* = 0.303). Similarly, whether the study size was large or small had little effect on the observed blood loss (*p* = 0.357). Meta-regression analysis further revealed that greater differences in hysterectomy rates between AABO and control groups were associated with larger reductions in blood loss (*p* = 0.018), whereas year of publication, localization, and baseline control hysterectomy rates were not statistically significant moderators. The model explained around 46% of the between-study variance (R^2^ = 45.7%). Funnel plot and Egger’s test (*p* = 0.70) suggested no strong evidence of small-study effects (Figure 4A).

Second, for hysterectomy rates, all 24 studies were included in the meta-analysis. The pooled log risk ratio showed a significant reduction in hysterectomy risk associated with AABO (log RR = −0.86, 95% CI: −1.24 to −0.49; *p* < 0.001), indicating that AABO is linked to lower hysterectomy rates. There was a notable variation in hysterectomy outcomes across the included studies (I^2^ = 68.8%). In the multivariable meta-regression, study sample size appeared to influence the observed effect (*p* < 0.001), with larger studies showing smaller effect sizes, suggesting a potential small-study effect. The baseline control hysterectomy rate in the control group also showed an association with the effect size (*p* = 0.033), as studies with higher baseline risk tended to report a stronger relative effect of AABO. By contrast, publication year did not appear to affect the results (*p* = 0.688), suggesting no clear temporal pattern.

The model explained approximately 75% of between-study variance (R^2^ ≈ 74.7%), and residual heterogeneity decreased to I^2^ ≈ 34%. A funnel plot showed mild asymmetry (Figure 4B); however, Egger’s test was not statistically significant (*p* = 0.098), indicating no strong evidence of publication bias.

#### 3.2.2. Complications

Comparison of complications in cases with cesarean section with AABO vs. cesarean section alone is presented in Table 3. The following complications were the most common: fever, bladder or ureteral injury, hemorrhagic shock, and lower limb arterial thrombosis. Notably, there was a difference in the rate of these complications between patients who had cesarean section with AABO vs. those who had cesarean section alone: hemorrhagic shock was seen in 3.8% of patients who had cesarean section with AABO vs. 14.6% among those who had cesarean section. Repeated surgery (re-laparotomy) was performed in 2.8% of women who had cesarean section with AABO vs. 8.7% of those who had cesarean section alone (Table 3). The rate of bladder and ureteral injury was also higher among women who had a cesarean section alone compared to the patients who had a cesarean section with AABO, 6.4% vs. 4.3%, respectively.

**Table 3:** Complications observed between groups.

Complications	CS + AABO (Number of Complications/Number of Patients in Studies)	CS Alone (Number of Complications/Number of Patients in Studies)	Studies Reporting the Complication
Hemorrhagic shock	10/265 (3.8%)	35/239 (14.6%)	[4,16,18,20,25]
Disseminated intravascular coagulation	5/409 (1.2%)	28/488 (5.7%)	[2,4,5,14,15,18,25]
Pulmonary embolism	2/542 (0.4%)	3/420 (0.7%)	[4,14,15,24]
Lower limb arterial thrombosis	41/1119 (3.7%)	0/940 (0%)	[2,3,4,5,8,9,10,13,15,18,21,24,25,26]
Lower limb vein thrombosis	15/912 (1.6%)	9/493 (1.8%)	[5,10,15,22,24,25,26]
Bladder or ureteral injury	36/846 (4.3%)	40/622 (6.4%)	[2,4,5,8,14,15,17,18,24,25,27]
Bowel injury	0/297 (0%)	1/98 (1%)	[18,24]
Intestinal obstruction	4/198 (2%)	6/140 (4.3%)	[4,15]
Neurological complications of the lower limbs	6/222 (2.7%)	0/187 (0%)	[2,5,10,16]
Infection	7/266 (2.6%)	8/334 (2.4%)	[4,8,10,14]
Fever	4/68 (5.9%)	7/65 (1%)	[2,15]
Local hematoma	6/572 (1%)	0/328 (0%)	[16,23,24,26]
Repeated surgery	27/951 (2.8%)	81/935 (8.7%)	[17,20,24,25,26,27,28]

Table footnotes: AABO—abdominal aorta balloon occlusion; CS—cesarean section.

#### 3.2.3. Neonatal Outcomes

Neonatal outcomes in cases of cesarean section with AABO and cesarean section alone are presented in Supplementary Table S2. All studies reported that the aortic balloon occlusion procedure is safe for a fetus. Out of 24 studies analyzed, 91.6% reported no difference in neonatal outcomes between cases where cesarean section was performed with AABO and alone (Supplementary Table S2). Only one study by Zhao et al. (2024) reported a lower admission rate to the neonatal intensive care unit in newborns whose mothers underwent cesarean section combined with AABO [27].

### 3.3. Risk of Bias

All 24 studies included in the analysis were non-randomized; therefore, they were assessed for bias according to the Newcastle–Ottawa Scale (NOS) for case–control studies [11]. Based on the assessment, 10 studies (41.7%) were scored as low risk of bias and 14 studies (58.3%) as moderate risk (Supplementary Table S3, Part A). The bias was mainly caused by the discrepancy in the measurement of the outcomes, some missing data, and the selection of the reported results.

Since all the studies that were included in the analysis were non-randomized observational retrospective studies, the certainty of evidence was considered to be low and then downgraded or upgraded based on the domains of the GRADE system (Supplementary Table S3, Part B).

### 3.4. Evaluation of the AABO Technique Trends

Analysis of publications through the period of observation (2015–2025) suggested an increasing level of improvement in efficacy and standardization of procedures over time, including an increase in the use of infrarenal placement and a multidisciplinary approach. Subsequent publications demonstrated a better decrease in blood loss and hysterectomy rates without an increase in complication rates. In particular, the infrarenal approach was standardized for more than 90% of studies, and the outcomes have been reported to improve over the years (Table 2 and Table 3). This may be attributed to better patient selection.

## 4. Discussion

As provided by the Society for Maternal-Fetal Medicine Placenta Accreta Spectrum Ultrasound Marker Task Force, the definition of placenta accreta spectrum disorders incorporates the terms placenta accreta, placenta increta, placenta percreta, morbidly adherent placenta, and invasive placentation [29]. It includes the full range of abnormal placental attachment to the uterus or other neighboring organs and is associated with severe obstetric hemorrhage that could result in hysterectomy, other severe complications, and maternal death [30]. Multiple interventions are proposed to decrease intraoperative bleeding, and, thus, improve maternal outcomes [1,31,32]. These techniques are widely used to decrease intraoperative hemorrhage during cesarean section for patients with PAS disorder. One of the most promising approaches is occlusion of the abdominal part of the aorta [1]. Thus, this study aimed to systematically assess the abdominal aorta balloon occlusion efficiency and safety in patients with PAS disorder.

This study analyzed the most recent research on the evaluation of outcomes after cesarean section accompanied by aortic balloon occlusion and cesarean section alone. Main outcomes such as blood loss, blood transfusion, hysterectomy, neonatal outcomes, and complication rates were compared between groups and analyzed across the studies included in this study.

Out of 24 studies analyzed in this systematic review and meta-analysis, in 22 investigations aortic occlusion was performed at the infrarenal level, while in one study [4] the blockage was at the level of Zone III of the aorta. One study did not specify the level of the aortic balloon occlusion. The infrarenal level, as the most common choice for the aorta occlusion, is justified by the anatomy of the blood vessels supplying the uterus and was found to be optimal [2,3,4,5,6,8,9,12,13,14,15,16,17,18,19,20,21,22,23,24,25,26,27,28].

The results of this study reveal that the blood loss in patients who underwent cesarean section with AABO was 1.8 times less than in cases with cesarean section alone. Additionally, analysis of the included studies’ outcomes clearly shows that the need for blood transfusion was lower in patients who underwent cesarean section with AABO. The rate of hysterectomies was three times higher among patients who had a cesarean section alone (without AABO). Furthermore, the rate of hemorrhagic shock was nearly four times higher if a cesarean section was performed for patients with PAS disorders without the assistance of AABO. Moreover, re-laparotomy was three times lower among women who had a cesarean section with AABO compared with those who had a cesarean section alone. These findings align with previous systematic reviews investigating the efficacy of aortic balloon occlusion in preventing adverse outcomes during cesarean delivery [33,34,35] and support the idea of utilization of the AABO procedure as a preventive measure, allowing for a decrease in maternal blood loss.

Our meta-analysis showed that the AABO procedure significantly reduced intraoperative blood loss, with patients who underwent AABO losing, on average, 933 mL less blood compared with controls (95% CI: 611–1255 mL; *p* < 0.001). This difference is clinically relevant, as reduced blood loss may lower the need for transfusion and facilitate surgical management. Heterogeneity across studies was very high (I^2^ = 98.65%), which may be due to differences in patient characteristics, surgical approaches, and perioperative care practices across the included studies. Subgroup analysis by balloon location and study size did not explain the variability. Meta-regression analysis demonstrated that greater reductions in blood loss were associated with larger differences in hysterectomy rates between groups (*p* = 0.018), while the year of publication and balloon location and baseline control hysterectomy rates were not statistically significant moderators. Even after considering these factors, heterogeneity remained high (I^2^ > 96%), suggesting that other unmeasured factors, such as surgical expertise, patient selection, or institutional transfusion protocols, may have contributed to the variability.

In addition to reducing blood loss, our analysis suggests that AABO performed during cesarean section may reduce the risk of hysterectomy, although the effect size varies across studies, likely influenced by study size and baseline risk. These findings should be interpreted cautiously, given that all included studies were retrospective.

This systematic review and meta-analysis provide an updated synthesis of current evidence regarding the effectiveness of the AABO procedure performed during cesarean section, and the results and conclusions of this study are instrumental for clinical practice, supporting the utilization of AABO for PAS disorder cases. Although results of this analysis suggest a potential benefit of AABO, the risk of severe complications related to AABO should be kept in mind while making decisions about its implementation. Such adverse events as pulmonary embolism, lower limb arterial and venous thrombosis, disseminated intravascular coagulation, neurological complications, etc., should not be underestimated. Thrombosis of the lower limb arterial vessels is considered one of the most significant vascular complications of AABO. Although it is not common, it highlights the importance of precise endovascular techniques, accurate balloon placement, careful monitoring of balloon occlusion time, and vascular monitoring in the postoperative period. Several studies included in the current analysis highlighted the importance of compliance with standard protocols during the preoperative and intraoperative periods, which may also minimize the chances of thrombotic/embolic episodes after AABO [2,3,4,5,8,9,10,13,15,18,21,24,25,26]. Thus, the role of AABO may be considered an important adjunct in the management of PAS, though it is imperative that this be performed within a multidisciplinary team, as well as monitoring for complications.

In addition to the AABO procedure, the management of complex cases of PAS requires comprehensive surgical management and the utilization of alternative and complementary techniques/measures in the control of hemorrhage [36,37]. The complex approach ensures better maternal outcomes. The alternative methods of bleeding control in the management of PAS complex cases include IIABO, UAE, uterine compression sutures (the B-Lynch suture; the Nausicaä suture), devascularization of the pelvis, Bakri balloon, and the option of planned cesarean hysterectomy [38,39,40,41,42]. Moreover, in recent years, new surgical techniques for hemorrhage control have been developed [40,41,42]. These new methods aim to improve the uterine compression and control of vascular bleeding and potentially could be useful in addition to AABO or used alone in those settings where the comprehensive approach with utilization of interventional radiology is not available.

Study Strengths and Limitations. This systematic review and meta-analysis provide a comprehensive and up-to-date and thorough synthesis of current evidence on the use of AABO in the management of PAS disorders. A key strength of the study lies in its rigorous methodological approach, including a comprehensive search across multiple databases, clear eligibility criteria, and structured assessment of bias using the Newcastle–Ottawa Scale. By summarizing maternal, surgical, and neonatal outcomes across a wide range of studies, this review offers clinically meaningful insights for obstetric teams managing high-risk pregnancies.

Another strength is the focus on both efficacy and safety outcomes, highlighting not only the potential of AABO to reduce blood loss, transfusion requirements, and hysterectomy rates, but also its associated complications. This dual perspective allows clinicians to make better-informed decisions about incorporating AABO into multidisciplinary management protocols.

However, several limitations must be acknowledged. All included studies were retrospective and non-randomized, which introduces potential selection bias and limits the strength of causal inferences. Unfortunately, most of the reports included in this study did not stratify PAS disorder subtypes for their original analysis; thus, this limitation did not allow a stratified meta-analysis. Moreover, since analysis of hemorrhagic shock was performed based on the data as reported by the original studies that have not followed a uniform definition, and the reporting units varied between the included studies, this may affect the comparability of the results. Variability in transfusion metrics among the original reports included in the analysis may affect comparability across studies and interpretation of the pooled findings. Additionally, incomplete reporting of some perioperative and long-term outcomes reduced the ability to fully evaluate safety profiles and neonatal effects. Finally, the predominance of studies conducted in a single geographic region limits the generalizability of findings to broader clinical settings and other healthcare systems. A major limitation of this review is the increased likelihood of selection bias, since all the trials included were retrospective in nature, and the application of AABO treatment was subjective. It is possible that the patients selected were either less sick or easier to treat, which would result in either an underestimate or overestimate of the effect of AABO treatment. Nevertheless, the review provides a valuable synthesis of current evidence and underscores the need for well-designed, multicenter prospective trials to confirm the benefits and further define the optimal role of AABO in PAS management. Future studies should report stratified outcomes by PAS subtype with a unified definition of hemorrhagic shock and transfusion units to better define which patient groups benefit most from AABO.

Despite the consistent observation from our findings that AABO application is associated with better maternal outcomes, such as reduced intraoperative blood loss, reduced transfusion rates, and hysterectomy rates, it should still be noted that these findings should be carefully interpreted due to their limitations as all the available studies conducted on the field were purely observational, mainly being retrospective in nature; hence, it would be inappropriate to assume cause and effect relationships between variables. 

## 5. Conclusions

Placental pathology remains a cause of major complications during pregnancy and delivery. It brings a high risk of heavy bleeding, hysterectomy, and maternal morbidity. The findings of this study indicate a consistent association between the use of AABO during cesarean delivery for women with PAS disorders and improved maternal outcomes, based on evidence of moderate-to-high certainty drawn from observational studies. Specifically, AABO is associated with a marked reduction in intraoperative blood loss, lower transfusion requirements, and a significantly decreased likelihood of hysterectomy and hemorrhagic shock, without compromising neonatal outcomes. While these results support the use of AABO as an effective adjunct in the surgical management of PAS, its implementation requires careful patient selection, experienced multidisciplinary teams, and institutional readiness. Potential complications, including thromboembolic events and vascular injuries, although infrequent, necessitate vigilant intraoperative and postoperative monitoring, as well as adherence to preventive measures. In clinical practice, AABO can be considered a valuable component of comprehensive PAS management strategies, complementing accurate prenatal diagnosis, preoperative planning, and coordinated surgical care. Nevertheless, the findings of this review should be cautiously interpreted due to the fact that all of the studies included in this analysis were observational and mostly retrospective in nature. Moreover, the existence of significant heterogeneity between studies and preponderance of data from one geographical location might influence the generalizability of results obtained from these studies. It is evident that even though AABO seems to be a useful additional treatment modality for PAS management, its therapeutic significance cannot be conclusively determined using the available evidence. Future well-designed prospective randomized multicenter studies are needed to refine patient selection criteria, standardize procedural protocols, and further evaluate the long-term maternal and neonatal safety of AABO.

## Data Availability

No new data were created or analyzed in this study.

## Supplementary Materials

The following supporting information can be downloaded at: https://www.mdpi.com/article/10.3390/jcm15093400/s1, Supplementary Table S1. Search threads; Supplementary Table S2. Neonatal outcomes among cases (cesarean section and AABO) vs. controls (cesarean section alone) Supplementary Table S3. Risk of bias in the included non-randomized studies. Supplementary files: PRISMA Checklist; PRISMA abstract checklist. Reference [43] is cited in Supplementary Materials.

## Abbreviations

AABO	Abdominal Aorta Balloon Occlusion
GRADE	Grading of Recommendations, Assessment, Development, and Evaluation
ICU	Intensive Care Unit
IIABO	Internal Iliac Artery Balloon Occlusion
IVF	In Vitro Fertilization
MeSH	Medical Subject Headings
NICU	Neonatal Intensive Care Unit
NOS	Newcastle–Ottawa Scale
PAS	Placenta Accreta Spectrum
PBOAA	Prophylactic Balloon Occlusion of Abdominal Aorta
PICO	Population, Intervention, Comparison, Outcomes
REBOA	Resuscitative Endovascular Balloon Occlusion of the Aorta
UAE	Uterine Artery Embolization
