# Supplementary material for: Assessment of Abdominal Aorta Balloon Occlusion Efficiency and Safety in Patients with Placenta Accreta Spectrum Disorder: A Systematic Review and Meta-Analysis
Source: J Clin Med. 2026 Apr 29;15(9):3400. doi: 10.3390/jcm15093400 (PMC13163405; doi:10.3390/jcm15093400)
Supplement: Supplementary file 1 [file jcm-15-03400-s001.zip › Supplementary/Supplementary Table S1.pdf]

Supplementary Table S1. Search threads

Concept	Terms
Population: Patients with placenta accreta spectrum disorder	placenta OR placenta accreta syndrome OR PAS OR morbidly adherent placenta OR MAP placenta accreta OR abnormally invasive placenta OR AIP OR placenta abnormality syndrome
Intervention: Abdominal aortic balloon occlusion	REBOA OR aortic balloon OR aortic vascular control OR prophylactic resuscitative endovascular balloon occlusion of the aorta OR intra-aortic balloon OR aortic occlusion OR abdominal aorta balloon occlusion OR AABO OR prophylactic aortic balloon occlusion OR ABO OR PAABO OR aortic vascular balloon control OR aortic vascular occlusion OR aortic vascular balloon occlusion OR PABO
Comparison: Standard cesarean section	cesarean section OR standard cesarean section OR cesarean section alone OR conventional management
Outcome: hysterectomy, blood loss, transfusion volume, ICU stay, APGAR scores, neonate weight	hysterectomy OR hysterectomy rate OR uterine preservation OR blood loss OR hemorrhage OR blood transfusion OR transfusion volume OR ICU stay OR APGAR OR APGAR score OR neonatal weight OR birth weight
