# Supplementary material for: Assessment of Abdominal Aorta Balloon Occlusion Efficiency and Safety in Patients with Placenta Accreta Spectrum Disorder: A Systematic Review and Meta-Analysis
Source: J Clin Med. 2026 Apr 29;15(9):3400. doi: 10.3390/jcm15093400 (PMC13163405; doi:10.3390/jcm15093400)
Supplement: Supplementary file 1 [file jcm-15-03400-s001.zip › Supplementary/Supplementary Table S2.pdf]

Supplementary Table S2. Neonatal outcomes among cases (cesarean section and AABO) vs. controls (cesarean section alone)

Author, year	Birth weight, g		APGAR score <7 at 1 min		APGAR score <7 at 5 min		NICU admission		Neonatal complications		Neonatal hospitalization, days or # of babies		Neonatal mortality	Conclusion
	case	control	case	control	case	control	case	control	case	control	case	control		
Chen et al., 2016	not reported	not reported	not reported	not reported	not reported	not reported	not reported	not reported	asphyxia 4 (20)	asphyxia 8 (34.8)	not reported	not reported	not reported	No difference in neonatal outcome between groups. Radiation 52.85.±11.3 mGy
Cui et al., 2017	2792.1±503.7	3042.2±573.6	1 (3)	4 (13)	0 (0)	1 (3)	not reported	not reported	not reported	not reported	4.6±4.2 days	5.3±4.3 days	not reported	No difference in neonatal outcome. outcome between groups. Radiation 3.3±1.1 mGy
Duan et al., 2018	2987±826	3195±735	Minutes not reported	Minutes not reported	Minutes not reported, mean score 9.6±0.8	Minutes not reported, mean score 9.5±0.9	not reported	not reported	asphyxia 0 (0)	asphyxia 2 (8.7)	not reported	not reported	0	No difference in neonatal outcome between groups, healthy babies in 2year follow up call. Radiation 4.1±3.2 mGy
Huo et al., 2021	2647.50±355.48	2580.31±503.58	8.83±1.52	9.81±0.75	9.75±0.45	9.94±0.25	not reported	not reported	not reported	not reported	not reported	not reported	not reported	Radiation exposure not reported
Ioscovich et al, 2023	2405.7±678.48	2315.45±605.48	5.9±3.13	6.27±2.57	7.4±1.64	7.63±1.8	not reported	not reported	not reported	not reported	not reported	not reported	not reported	No difference in neonatal outcome b/w groups. No radiation,

														ultrasound guidance
Li et al., 2018	2928.75±411.776	2698.03±652.810	8.79±1.644	8.47±1.852	9.48±0.790	9.40±0.932	not reported	not reported	not reported	not reported	6 (25) babies	14 (43.8)	2 neonates in control group (1- 26w old, 2- congenital malformations)	No difference in neonatal outcome between groups. Radiation exposure not reported
Liu et al., 2021	2909.07±381.764798	2975.47±425.647	8.36±1.255	8.73±1.676	9.61±0.674	9.71±0.804	not reported	not reported	asphyxia 11 (6.5)	asphyxia 11 (10.4)	not reported	not reported	0	No difference in neonatal outcome between groups. Radiation exposure less than 10 mGy (teratogenic after 100mGy and more)
Liu et al., 2022	2611.50±365.20	2678.00±328.26	2 (5)	0 (0)	not reported	not reported	not reported	not reported	not reported	not reported	not reported	not reported	not reported	No difference in neonatal outcome between groups. Fetal radiation exposure in balloon group = 5.05±1.08 mGy
Lu et al., 2021	2875 (2484-3163)	2800 (2400-3200)	12 (18.2)	37 (19.1)	8 (12.1)	20 (10.3)	23 (34.8)	87 (44.8)	not reported	not reported	not reported	not reported	case – 6 (9.1), control – 13 (6.7)	No comments from authors, from tables data – no difference, don't report on radiation exposure
Luo et al., 2021	2768.8±502.4	2958±602.5	not reported	not reported	median (interquartile range) 10 (9-10)	10 (10)	not reported	not reported	not reported	not reported	not reported	not reported	not reported	No difference in neonatal outcome

														between groups. Radiation exposure not reported
Luo et al., 2022	2645±464	2602±470	8.1±1.3	8.2±1.3	9.4±0.8	9.5±0.8	not reported	not reported	not reported	not reported	not reported	not reported	not reported	No difference in neonatal outcome between groups. Radiation exposure not reported
Mei et al., 2022	2700 (2200-2860)	2700 (2500-2980)	not reported	not reported	not reported	not reported	not reported	not reported	not reported	not reported	not reported	not reported	not reported	Radiation exposure is 1.9±0.4 mGy
Sun et al., 2018	2839±443	2955±514	10	10	10	10	not reported	not reported	not reported	not reported	not reported	not reported	not reported	No difference in neonatal outcome between groups. Foetal radiation exposure in balloon group = 4.2±1.49 mGy
Wang et al., 2017	not reported	not reported	1 (10)	1 (3.0)	0	0	not reported	not reported	not reported	not reported	not reported	not reported	1 in control group – severe malformations, excluded from APGAR scoring	No difference in neonatal outcome between groups. Radiation exposure not reported, but say in methods its less than 1 min and 50 mGy
Wang et al., 2022	not reported	not reported	not reported	not reported	not reported	not reported	not reported	not reported	not reported	not reported	not reported	not reported	not reported	No data on neonatal outcomes or radiation exposure

Wang et al., 2023	2867.79±398.44	2766.19±796.06	9	9	10	10	not reported	not reported	not reported	not reported	not reported	not reported	not reported	No difference in neonatal outcome between groups. Radiation 6.17 mGy (range 3.02-8.91)
Wu et al., 2016	2700±200 they provide data in kg	2600±200	8.8±0.9	8.4±0.9	9.7±0.5	9.6±0.6	not reported	not reported	not reported	not reported	not reported	not reported	0 in both groups	No difference in neonatal outcome between groups. radiation exposure in balloon group = 5.1±3.0 mGy
Xie et al., 2017	not reported	not reported	not reported	not reported	<4 - 0	<4 - 0	5 (17.2)	7 (17.5)	not reported	not reported	not reported	not reported	2 stillbirths in case and 2 stillbirths in control group (cause not reported)	No difference in neonatal outcome between groups. Radiation exposure not reported, but say in discussion its less than 50 mGy
Ye et al., 2023	2805 (290) mean, SD	2890 (323)	8.87 (1.15)	8.58 (1.41)	9.57 (0.81)	9.35 (1.36)	176 (63.3)	52 (60.4)	not reported	not reported	not reported	not reported	not reported	No difference in neonatal outcome between groups. Radiation exposure 2.3 (1.0) mGy
Yin et al., 2022	2778.75±543.86	2617.40±594.53	19 (27.94)	6 (6.82)	2 (2.94)	2 (2.27)	45 (66.18)	46 (52.27)	asphyxia 19 (27.94) infection 5 (7.35)	asphyxia 6 (6.82) infection 13 (14.77)	8.67±7.43	9.24±8.85	not reported	No difference in neonatal outcome between groups.

														Radiation exposure not reported
Zeng et al., 2017	2704.17±518.42	2595.95±510.61	7.60±1.23	7.81±0.94	9.4±0.82	9.32±0.78	8 (16.7)	6 (15.8)	not reported	not reported	not reported	not reported	not reported	No difference in neonatal outcome between groups. Radiation exposure not reported
Zheng et al., 2019	not reported	not reported	not reported	not reported	10 (9-10)	10 (10)	9 (8.8)	12 (18.5)	not reported	not reported	not reported	not reported	7 – termination of pregnancy in 2nd trimester, 1 – antenatal death at 36w – all excluded from APGAR scoring	No difference in neonatal outcome between groups. Radiation exposure 3.2 mGy (0.2-71)
Zhao et al., 2024	2960±1340	2640±1130	8.13±1.24	8.32±1.16	9.15±1.68	9.26±1.25	14 (11.86)	7 (31.82)	10 (8.47)	7 (31.82)	not reported	not reported	not reported	NICU admission was lower in AABO group
Zheng et al., 2022	2693.98±604.64	2699.13±655.38	8.67±1.79	8.53±1.68	9.43±1.55	9.53±1.26	not reported	not reported	not reported	not reported	not reported	not reported	not reported	No difference in neonatal outcome between groups. Radiation exposure not reported, but also don't say if US-guided or angiography

Table footnotes: NICU – neonatal intensive care unit; AABO – abdominal aorta balloon occlusion
