# Supplementary material for: Assessment of Abdominal Aorta Balloon Occlusion Efficiency and Safety in Patients with Placenta Accreta Spectrum Disorder: A Systematic Review and Meta-Analysis
Source: J Clin Med. 2026 Apr 29;15(9):3400. doi: 10.3390/jcm15093400 (PMC13163405; doi:10.3390/jcm15093400)
Supplement: Supplementary file 1 [file jcm-15-03400-s001.zip › Supplementary/Supplementary Table S3.pdf]

Supplementary Table S3. Risk of bias in the included non-randomized studies

Part A. Assessment of included studies

Study, year	Study design	Selection (0-4)	Comparability (0-2)	Outcome/exposure (0-3)	Total score (0-9)	Risk of bias*
Chen et al., 2016	Retrospective	3	1	2	6	Moderate
Cui et al., 2017	Retrospective	3	1	2	6	Moderate
Duan et al., 2018	Retrospective	3	1	2	6	Moderate
Huo et al., 2021	Retrospective	3	1	2	6	Moderate
Ioscovich et al., 2023	Retrospective cohort	4	1	2	7	Low
Li et al., 2018	Retrospective	3	1	2	6	Moderate
Liu et al., 2021	Retrospective cohort	4	2	2	8	Low
Liu et al., 2022	Retrospective case–control	3	1	2	6	Moderate
Lu et al., 2021	Retrospective	3	1	2	6	Moderate
Luo et al., 2021	Retrospective case–control	3	2	2	7	Low
Luo et al., 2022	Retrospective case–control	3	1	2	6	Moderate
Mei et al., 2022	Retrospective observational	3	1	2	6	Moderate
Sun et al., 2018	Retrospective	3	1	2	6	Moderate
Wang et al., 2017	Prospective cohort	4	2	2	8	Low
Wang et al., 2022	Retrospective cohort	4	2	2	8	Low
Wang et al., 2023	Retrospective case–control	3	1	2	6	Moderate
Wu et al., 2016	Retrospective cohort	4	1	2	7	Low
Xie et al., 2017	Retrospective case–control	3	1	2	6	Moderate

Ye et al., 2023	Retrospective cohort	4	2	2	8	Low
Yin et al., 2022	Retrospective	3	1	2	6	Moderate
Zeng et al., 2017	Retrospective cohort	4	1	2	7	Low
Zhao et al., 2024	Retrospective cohort	4	1	2	7	Low
Zheng et al., 2019	Retrospective case–control	3	1	2	6	Moderate
Zheng et al., 2022	Retrospective case–control	3	2	2	7	Low
Table footnotes<br>*Risk of bias was classified as: Low risk: ≥7; Moderate risk: 5–6						

Part B. GRADE summary of certainty of evidence for main outcomes

**Outcome**	**Study design**	**Risk of bias**	**Inconsistency**	**Indirectness**	**Imprecision**	**Publication bias**	**Overall certainty (GRADE)**
Intraoperative blood loss	Observational (retrospective / cohort)	Not serious	Not serious	Not serious	Not serious	Undetected	Moderate
Blood transfusion requirement	Observational	Not serious	Not serious	Not serious	Not serious	Undetected	Moderate
Hysterectomy rate	Observational	Not serious	Not serious	Not serious	Not serious	Undetected	Moderate
Maternal complications	Observational	Not serious	Serious (heterogeneous definitions and reporting)	Not serious	Not serious	Undetected	Low to moderate
Neonatal outcomes	Observational	Not serious	Serious (limited and heterogeneous reporting)	Serious (incomplete radiation and long-term data)	Serious	Undetected	Low
